# Risk factors for dementia in the epidemiological study of Munguialde County (Basque Country-Spain)

**DOI:** 10.1186/1471-2377-8-39

**Published:** 2008-10-15

**Authors:** Manuel Fernández Martínez, Jessica Castro Flores, Susana Pérez de las Heras, Aitziber Mandaluniz Lekumberri, María Gordejuela Menocal, Juan José Zarranz Imirizaldu

**Affiliations:** 1 Department of Neurology, Hospital de Cruces, Baracaldo, Vizcaya, Spain; 2 Department of Neurology, Hospital de Cruces, BBVA-Carolina Foundation Grant. Baracaldo, Vizcaya, Spain; 3 Llodio Health Centre, OROITU Medical Day Care Centre, Getxo, Vizcaya, Spain; 4 San Jose Etxealai Foundation, Munguia, Vizcaya, Spain

## Abstract

**Background:**

Prevalence of degenerative dementias and dementias associated with cerebrovascular disease is increasing. Dementia is one of the most significant public health problem. In recent years, the role of vascular risk factors (hypertension, diabetes mellitus and hypercholesterolemia) and depression has been evaluated.

The incidence of dementia and risk factors has not been fully investigated in Spain. The aim of this study was to identify the risk factors for dementia, Alzheimer's disease (AD) and vascular dementia (VD) in elderly people in Munguialde County (Spain).

**Methods:**

A two phase, door-to-door populational study was performed. Demographic variables and the presence of vascular risk factors and depression were recorded. The MMSE, the DSM-IV and the conventional criteria for AD and VD were used in the evaluation. The odds ratio for each risk factor was calculated by logistic regression analysis.

**Results:**

1756 healthy subjects and 175 patients with dementia participated in the study. Of these, 133 had AD, 15 VD and the remainder other dementias. The risk factors for dementia and AD were female sex (OR = 1.67 and 1.97, respectively); age (OR = 1.14 and 1.15); stroke (OR = 7.84 and 3); and depression (OR = 53.08 and 3.19). Stroke was the only risk factor for VD (OR = 119).

**Conclusion:**

Greater age, female sex, stroke and depression increase the risk of suffering dementia, AD and VD. The relationship between depression, vascular risk factors and dementia has clear public health implications. Prevention and early treatment of vascular risk factors and depression may have an important impact in lowering the risk of dementia and could modify the natural history of the disease.

## Background

The prevalence of degenerative dementias and those associated with cerebrovascular disease is increasing due to the ageing of the population. Thus, dementia is one of the most significant public health problems. The World Health Organization calculates that there will be 29 million people worldwide affected by dementia by the year 2020, and that two thirds of these cases will be due to AD [1]. Moreover defining the prevalence of dementia is important to identify the risk factors in order to develop preventive measures that could modify its course.

Most studies on the risk factors for dementia have focused more closely on AD as this is the most common cause. Age, female sex and low educational level are factors that increase the incidence and prevalence of dementia and, specifically, of AD [2-4]. In recent years, the role of the vascular risk factors (hypertension, type 2 diabetes mellitus and hypercholesterolemia) has been included in this evaluation. The existence of a vascular component that reduces cerebral perfusion has been postulated in AD [3]. The vascular component could participate in the neurodegenerative process intensifying the symptoms of the disease [5]. Recent studies [6,7] have suggested that depression is a risk factor for AD, but the basis for this association is unclear.

A recent study [8] has revealed that female sex, advanced age, depression and intake of vitamine supplements were independent related factor for AD, whereas depression and low-density lipoprotein-cholesterol (LDL-C) were independent related factor for VD.

There are few studies [9-12] that have evaluated the presence of these risk factors in the Spanish population. Our objective has been to identify the risk factors for dementia, Alzheimer's disease (AD) and vascular dementia (VD) in elderly people of Mungialde County (Vizcaya-Basque Country).

## Methods

### Study population

The study included all persons aged 65 years and over with registered residence in the county of Munguialde in January 2005.

A database into which all the information was entered was designed in Access 2003. This database was declared to the Data Protection Agency. The Ethics Committee of Cruces Hospital supervised and approved the study design, the ethical considerations, the confidentiality of the information and the informed consent for the participants.

### Study design

A two phase, door-to-door population-based study was performed between January and December, 2005[13,14]. The first phase (screening phase) included an evaluation of the cognitive status using the MMSE (Minimental State Examination) [15] and the Pfeiffer questionnaire (SPMSQ) [16]. Individuals with a score of less than 24 points were considered positive and went to the following phase. In illiterate individuals additionally to MMSE, those with scores of over 4 in the SPMSQ were included.

In the second phase (diagnostic confirmation phase), the subjects suspected of having dementia were evaluated by a doctor (neurologist, geriatrician or family doctor) using a structured history and clinical examination that included the Clinical Dementia Rating scale (CDR scale). Behaviour was evaluated using the Neuropsychiatric Inventory [17]. Furthermore, two independent neuropsychologists performed the MMSE and the seven-minute test (7MT) [18].

### Diagnosis of dementia

Finally, the principal investigator (MFM) made the diagnosis of dementia based on the DSM-IV criteria [19], the NINCDS-ADRDA criteria for AD [20] and the NINCDS-AIREN criteria [21] and the Hachiski scale [22] for VD.

### Evaluation of the risk factors

The information on the risk factors was gathered in the first phase. Specifically designed questions were asked for each one of the following risk factors: sex, age (years of age at the time of the interview) and education (years of education completed). The subjects were evaluated by a doctor using a structured interview of the subject's medical history. The diagnoses of hypertension, hypercholesterolemia, stroke, diabetes mellitus and depression were based on the diagnoses referred by the patient or carer, a review of the clinical history and the use of specific medication. If the patient was suffering from heart disease, information about ischaemic heart disease, arrhythmias and heart failure was obtained from the patient or carer and from a review of the clinical history and the use of medication.

### Statistical analysis

The SPSS version 12.0.1 for Windows and G-Stat version 2.1 (GSK, Madrid) were used for the statistical analysis. Binary variables were created for each of the risk factors (for example, hypertension yes/no). The frequencies were calculated for each of the risk factors (hypertension, stroke, diabetes, hypercholesterolaemia, heart disease and depression) in controls and in patients with dementia, AD and VD.

Logistic regression was used to evaluate the association between risk factors and dementia. An initial, univariate model was therefore created. In this model each one of the risk factors (age, sex, and years of education, hypertension, stroke, diabetes, hypercholesterolemia, heart disease and depression) were included separately. Age and education were continuous variables. The reference group for each risk factor was the presence of disease. A second model was subsequently created. In the second model all the risk factors (age, sex, and years of education, hypertension, stroke, diabetes, hypercholesterolemia, heart disease and depression) were included simultaneously to determine the independent effect of each one of them. The same logistic regression analysis was performed in patients with AD and VD.

Finally, the patients were stratified into three groups according to the presence of vascular risk factors: no risk factor, one risk factor and more than one risk factor. A logistic model was created including each one of the risk groups, adjusting for age, sex, years of education and depression.

## Results

1756 healthy subjects and 175 patients with dementia participated in the study. Of the demented patients, 133 had AD, 15 VD and the remainder other dementias. Table 1 shows the sociodemographic and clinical characteristics of the study population. The group of patients with dementia was characterised by being of female sex, older and with fewer years of education. The frequency of each one of the risk factors is shown in table 2.

**Table 1:** Characteristics of the population with and without dementia

**Characteristics**	Subjects with dementia N = 175	Healthy subjects N = 1756	p
**Age **(mean ± SD)	81.58 (8.15)	73.95 (6.3)	< 0.001
Sex*			
Female	127 (72.6%)	950 (54.1%)	< 0.001
Male	48 (27.4%)	806 (45.9%)	< 0.001
**Age group**			
65–69*	10 (5.7%)	501 (28.5%)	< 0.001
70–74*	30 (17.1%)	542 (30.9%)	< 0.001
75–79	31 (17.7%)	364 (20.7%)	0.35
80–84*	34 (19.4%)	217 (12.4%)	0.01
≥ 85*	70 (40%)	132 (7.5%)	< 0.001
**Years of education **(SD) *	4.45 (3.85)	6.18 (3.99)	< 0.001
Educational level*			
Illiterate	76 (43.4%)	445 (25.3%)	< 0.001
Primary-Secondary	95 (54.3%)	1198 (68.2%)	< 0.001
Higher	4 (2.3%)	113 (6.4%)	0.03

**MMSE **(SD) *	12.61 (8.31)	27.22 (2.38)	< 0.001

*Statistically significant differences between the two groups.

SD: Standard deviation.

MMSE: Minimental State Examination

**Table 2:** Frequency of the risk factors in the study population

**Characteristic**	Dementia N = 175	AD N = 133	VD N = 15	Healthy Subjects N = 1756	P value. Dementia Versus Healthy Subjects
**Sex**					
Female	127(72.6%)	101(75.9%)	10(66.7%)	950(54.1%)	< 0.001
Male	48 (27.4%)	32 (24.1%)	5 (33.3%)	806(45.9%)	< 0.001
**Age group**					
65–69	10 (5.7%)	7 (5.3%)	1 (6.7%)	501(28.5%)	< 0.001
70–74	30 (17.1%)	19 (14.3%)	4 (26.7%)	542(30.9%)	< 0.001
75–79	31 (17.7%)	24 (18%)	3 (20%)	364(20.7%)	< 0.001
80–84	34 (19.4%)	23 (17.3%)	6 (40%)	217(12.4%)	< 0.001
≥ 85	70 (40%)	60 (45.1%)	1 (6.7%)	132 (7.5%)	< 0.001
**Educational level**					
Illiterate	76 (43.4%)	57 (42.9%)	8 (53.3%)	445(25.3%)	<0.001
Primary-Secondary	95 (54.3%)	74 (55.6%)	6 (40%)	1198(68.2%)	<0.001
Higher	4 (2.3%)	2 (1.5%)	1 (6.7%)	113 (6.4%)	<0.001
**Risk factors**					
Hypertension	63 (36%)	46 (34.6%)	6 (40%)	693(39.5%)	0.21
Stroke	46 (26.3%)	19 (14.3%)	12 (80%)	57 (3.2%)	<0.001
Diabetes	32 (18.3%)	22 (16.5%)	4 (26.7%)	213(12.1%)	0.02
Hypercholesterolaemia	21 (12%)	16 (12%)	2 (13.3%)	236(13.4%)	0.34
Heart disease	35 (20%)	26 (19.5%)	3 (20%)	294(16.7%)	0.16
Depression	14 (8%)	10 (7.5%)	1 (6.7%)	50 (2.8%)	< 0.001

**Vascular Risk Factors**					
None	59 (33.7%)	52 (39.1%)	2 (13.3%)	738(42%)	0.02
One	66 (37.7%)	48 (36.1%)	6 (40%)	640 (36.4%)	0.39
More than one	50 (28.6%)	33 (24.8%)	7 (46.7%)	378 (21.5%)	0.02

AD: Alzheimer's disease.

VD: vascular dementia

In the univariate model, female sex (OR = 2.24; 95% IC: 1.59–3.17; p < 0.001), years of education (OR = 1.13; IC: 1.08–1.19; p < 0.001), stroke (OR = 10.63; 95% IC: 6.93–16.30; p < 0.001), diabetes (OR = 1.62; IC: 1.07–2.44; p = 0.02) and depression (OR = 2.96; 95% IC: 1.61–5.48; p < 0.001) were risk factors for dementia. The youngest individuals had a lower risk (OR = 0.87; 95% IC: 0.85–0.89; p < 0.001) (table 3). In the second model (table 3), female sex (OR = 1.67; 95% IC: 1.14–2.45; p = 0.01), age (OR = 1.14; 95% IC: 1.11–1.17; p < 0.001), stroke (OR = 7.84; 95% IC: 4.80–12.81; p < 0.001) and depression (OR = 3.08; 95% IC: 1.50–6.29; p = 0.02) were independent risk factors for dementia. The years of education and diabetes were factors that did not reach statistical significance in this model.

**Table 3:** OR and 95% confidence intervals for total dementia, AD and VD vs. controls

	Model 1 univariate			Model 2 multivariate		
	
	Beta	OR (95% CI)	P	Beta	OR (95% CI)	p
**Dementia**						
Female sex	0.81	2.24(1.59–3.17)	< 0.001	0.52	1.67(1.14–2.45)	0.01
Age	-0.14	0.87(0.85–0.89)	< 0.001	0.13	1.14(1.11–1.17)	< 0.001
Years of education	0.13	1.13(1.08–1.19)	< 0.001	-0.04	1.04(0.99–1.09)	0.11
Hypertension	-0.15	0.86(0.62–1.19)	0.37	-0.32	0.72(0.50–1.06)	1
Stroke	2.36	10.63(6.93–16.30)	<0.001	2.06	7.84(4.80–12.81)	< 0.001
Diabetes	0.48	1.62(1.07–2.44)	0.02	0.48	1.61(0.99–2.60)	0.53
Hypercholesterolaemia	-0.13	0.87(0.54–1.41)	0.59	-0.06	0.94(0.53–1.64)	0.82
Heart disease	0.22	1.24(0.84–1.84)	0.27	-0.22	0.80(0.5–1.26)	0.33
Depression	1.08	2.96(1.61–5.48)	< 0.001	1.12	3.08(1.50–6.29)	0.02
**AD**						
Female sex	0.99	2.68 (1.78–4.03)	< 0.001	< 0.001	1.97(1.27–3.06)	< 0.001
Age	-0.15	0.86 (0.84–0.88)	< 0.001	< 0.001	1.15(1.12–1.18)	< 0.001
Years of education	0.13	1.14 (1.08–1.2)	< 0.001	< 0.001	0.96(0.91–1.01)	0.12
Hypertension	-0.21	0.81 (0.56–1.17)	0.26	0.26	0.72(0.48–1.09)	0.12
Stroke	1.60	4.96 (2.86–8.63)	< 0.001	< 0.001	3(1.58–5.67)	< 0.001
Diabetes	0.36	1.44 (0.88–2.32)	0.14	0.14	1.53(0.89–2.62)	0.12
Hypercholesterolaemia	-0.13	0.88 (0.51–1.51)	0.64	0.64	1.04(0.57–1.92)	0.88
Heart disease	0.19	1.21 (0.77–1.88)	0.41	0.41	0.82(0.5–1.34)	0.43
Depression	1.02	2.77 (1.37–5.6)	< 0.001	< 0.001	3.19(1.46–6.98)	< 0.001

**VD**						
Female sex	0.53	1.7 (0.58–4.99)	0.34			
Age	-0.09	0.92 (0.86–0.98)	< 0.001			
Years of education	0.15	1.16 (0.99–1.36)	0.06			
Hypertension	0.02	1.02 (0.36–2.88)	0.97			
Stroke	4.78	119 (32.74–434)	< 0.001			
Diabetes	0.96	2.63 (0.83–8.34)	1			
Hypercholesterolaemia	-0.01	0.99 (0.22–4.42)	1			
Heart disease	0.22	1.24 (0.34–4.43)	0.74			
Depression	0.89	2.43 (0.31–18.89)	0.39			

Model 1: univariate model

Model 2: multivariate model including demographic variables (age, sex and years of education), depression and vascular risk factors

OR: odds ratio

CI: confidence interval

AD: Alzheimer's disease

VD: vascular dementia

In AD, female sex (OR = 2.68; 95% IC: 1.78–4.03; p < 0.001), years of education (OR = 1.14; 95% IC: 1.08–1.2; p < 0.001), stroke (OR = 4.96; 95% IC: 2.86–8.63; p < 0.001), and depression (OR = 2.77; 95% IC: 1.37–5.6; p < 0.001) were risk factors in the univariate model. The youngest individuals had a lower risk (OR = 0.86; 95% IC: 0.84–0.88; p < 0.001) (table 3). In the second model, female sex (OR = 1.97; 95% IC: 1.27–3.06; p < 0.001), age (OR = 1.15; 95% IC: 1.12–1.18; p < 0.001), stroke (OR = 3; 95% IC: 1.58–5.67; p < 0.001) and depression (OR = 3.19; 95% IC: 1.46–6.98; p < 0.001) were independent risk factors for AD. The years of education lost statistical significance in this model (table 3).

In VD, neither sex nor the years of education showed an effect in the univariate model. The youngest individuals had a lower risk (OR = 0.92; 95% IC: 0.86–0.98; p < 0.001) (table 3). Stroke was the only risk factor in the univariate model (OR = 119; 95% IC: 32.74–434; p < 0.001)

Finally, the presence of more than one vascular risk factor increase the risk for dementia and VD (OR = 1.66; 95% IC: 1.06–2.58; p = 0.03 and OR = 7.77; 95% IC: 1.55–39.03; p = 0.01 respectively). In both groups, the absence of risk factors was protective (OR = 0.63; 95% IC: 0.40–0.97; p = 0.04 and OR = 0.13 95% IC: 0.03–0.65; p = 0.01 respectively) (table 4).

**Table 4:** Logistic regression analysis for the vascular risk groups

	beta	OR (95% CI)	p
**Dementia**			
None	-0.47	0.63 (0.40–0.97)	0.04
One	0.28	1.32 (0.90–1.95)	0.16
More than one	0.50	1.66 (1.06–2.58)	0.03
**AD**			
None	-0.17	0.84 (0.51–1.39)	0.51
One	0.67	1.07 (0.70–1.64)	0.76
More than one	0.17	1.19 (0.72–1.96)	0.51

**VD**			
None	-2.04	0.13 (0.03–0.65)	0.01
One	1.22	3.39 (0.68–16.88)	0.14
More than one	2.05	7.77 (1.55–39.03)	0.01

*model adjusted to demographic variables (age, sex, years of education) and depression.

OR: odds ratio

CI: confidence interval

AD: Alzheimer's disease

VD: vascular dementia

## Discussion

The objective of this study has been to evaluate the relationship between the different risk factors (demographic, vascular and depression) and dementia.

Most of the patients with dementia in our study were women (72.6%). Female sex was an independent risk factor for dementia and AD. In VD, female sex did not reach statistical significance. Some authors [23-25] have demonstrated that dementia and specifically, AD are more common in women. However, others [26,27] have not found differences between genders, and when it occurs is at very advanced ages (over 90 years of age) [28-31]. The risk of VD was similar in the two sexes, or slightly higher in men [28]. There could be a number of explanations for this finding: 1) in general, women live longer and they therefore have a higher probability of developing dementia; 2) the loss of the neuroprotective effect of the oestrogens would increase the prevalence of dementia in older women [4]; 3) Probably men reaching more advanced ages are an survival elite with more resistance to the risk factors [31].

Age increases the risk of dementia. Between 65 and 85 years of age, the prevalence doubles every 5.2 years, following an exponential model [2,23,26,27,32-36]. However, controversy exists about whether age has the same influence on AD and VD. Several studies [2,29,31,37] have shown that the increase in dementia with age occurs due to patients with AD. Our study supports this hypothesis as age was a risk factor for dementia and AD but not for VD.

In some studies [25,34,38] a lower educational level was a risk factor for the onset of dementia, though this has not been confirmed in other studies [39]. In a recent meta-analysis [40], the relative risk (RR) for patients with a lower educational level was 1.8 for AD (95% CI: 1.43–2.27) and was not significant for the other dementias. There are a number of hypotheses to explain the relationship between the years of education and dementia: 1) education may affect the results of some screening tests such as the MMSE, leading to an overestimation of the diagnosis of dementia in illiterate populations [40]; 2) a higher educational level would delay the clinical expression of dementia. The "cognitive reserve" hypothesis postulates that a higher educational level would increase neuronal plasticity and connectivity. In our study, we have used a large battery of tests and scales: MMSE, CRD, the seven-minute test and the SPMSQ. We consider that this strategy will have reduced the number of false positives among the population with fewer years of education. In the univariate analysis, the years of education represented a risk factor for dementia and AD. However, the strength of this association was lost in the multivariate analysis. The risk of VD in the illiterate population was not significant.

In our patients, depression was an independent risk factor for dementia, AD and VD. Other authors [7,41] have also demonstrated a significant association between depression and AD. In a meta-analysis [6], a history of depression doubled the risk for developing dementia and AD. Depression could produce degenerative lesions in the hippocampus mediated by the excess of glucocorticoids [42], neuronal loss in the aminergic nuclei of the brainstem [43] and a fall in the levels of noradrenaline and serotonin in the cerebral cortex and hippocampus [44]. Some cases of depression in the elderly could be secondary to cerebrovascular disease [45].

Stroke increase the risk of cognitive deterioration and AD by three- to six-fold [46,47], and by four- to nine-fold for VD [48], particularly if other vascular risk factors were present [47]. Our patients with stroke had a higher risk of dementia, AD and VD. The association between AD and stroke could be explained by a systemic vascular process (generalised atherosclerosis) [49], the additive effect of stroke on AD [50], or oligaemia that would intensify the amyloid cascade [5,51]. The presence of cerebrovascular disease intensifies the severity of AD symptoms [5] and leads to an earlier onset of symptoms [47]. In one study, the RR for VD associated with a history of stroke was 3.83 [52], and the clinical characteristics of the stroke and of the other vascular risk factors had an influence on the presence of dementia. In another cohort of 1301 patients older than 75 [53], the RR of incident VD associated with a history of stroke was 1.7, particularly when the stroke had occurred in the previous 3 years. In our study, the OR for stroke was high. This result could be explained by the few cases of VD identified and the high percentage of stroke (80%) in these patients.

The vascular risk factors (hypertension, hypercholesterolemia, heart disease and diabetes mellitus) are also risk factors for dementia and AD [54]. Earlier cross-sectional studies and follow-up studies yielded conflicting results on the relationship among hypertension, hypercholesterolemia and the onset of dementia. In some of them [2,55], there was an increase in the risk of dementia and AD whilst in others [56,57] its effect appeared to be small.

Our results did not find any relationship between vascular risk factors and AD, probably due to the small sample of patients or the cross-sectional design of the study. A recent cross-sectional study [8] that evaluated 1436 patients did not find any relationship either.

Recently, a number of longitudinal studies [58-60] have shown that hypertension, hypercholesterolemia and, particulary, their combination (OR = 2.8), in the middle ages of life; increase the risk of developing AD in later life. Explanations for these associations include: the coincidence of common disorders in the elderly; vascular and cerebrovascular disease precipitating AD; and an additive or synergistic (AD + vascular) pathogenesis of dementia.

In spite of vascular risk factors have a negative effects on cognition, the mechanisms linking these factors to AD remains uncler.

In some studies, diabetes increased the risk of dementia and VD [61], especially when associated with hypertension [62] or with heart disease [63]. In other studies, the risk of AD was increased [64]. In the Canadian study of health and ageing [65], no association was found between AD and diabetes. In our study, diabetes was the only risk factor that showed a statistical significance for dementia in the univariate analysis. However, diabetes mellitus lost statistical significance in the multivariate analysis. The other factors were not associated with any type of dementia.

In our patients the presence of two or more risk factors increased the OR for dementia and VD. The absence of these risk factors was protective. The presence of two or more vascular risk factors showed a tendency to increase the risk of AD, though this did not reach statistical significance. In a recent study [66], the RR for AD in the presence of three or more vascular risk factors was 3.4 on comparison with those individuals without risk factors.

The strengths of our study are that the evaluated population is large and representative of the Basque country. Furthermore, demographic and clinical variables have been evaluated togheter to determine the independent and combined effect of each one. On the other hand, a broad battery of test has been used in this study.

The main limitation is that the information is based on clinical records or on the presence of a specified risk factor. Furthermore, we have identified a few patients with VD, which could bias the results in some way. Finally, we consider that the next step should be a longitudinal study in the same population.

## Conclusion

In conclusion, our results suggest that additionally to age and female sex, a history of stroke and depression are independent risk factors for dementia and AD. Stroke is the only independent risk factor for VD. Multiple vascular risk factors increases the probability for developing any type of dementia. The relationship between stroke, depression, vascular risk factors and dementia has clear implications for the public health.

## Competing interests

The authors declare that they have no competing interests.

## Authors' contributions

MFM main investigator, conceived of the study, and participated in its design and coordination. JCF co-investigator; participated in its design and coordination. SPH co-investigator; participated in its design and coordination. AML participated in the design of the study, performed the statistical analysis. MGM participated in the design of the study, performed the statistical analysis. JJZ drafted the manuscript. All authors read and approved the final manuscript.

## Pre-publication history

The pre-publication history for this paper can be accessed here:


